# Prevalence of hypertension and other cardiovascular disease risk factors among university students from the National Polytechnic Institute of Côte d’Ivoire: A cross-sectional study

**DOI:** 10.1371/journal.pone.0279452

**Published:** 2023-01-05

**Authors:** Philippe C. Zobo, Frank Y. Touré, Iklo Coulibaly, Alexandra M. Bitty-Anderson, Simon P. Boni, Serge Niangoran, Annick Guié, Hermann Kouakou, Boris Tchounga, Patrick A. Coffie, Didier K. Ekouevi

**Affiliations:** 1 Heart of Grace Foundation, Abidjan, Côte d’Ivoire; 2 PACCI Program, Abidjan, Côte d’Ivoire; 3 Department of Cardiology and Cardiothoracic Surgery, Training and Research Unit of Medical Sciences, Félix Houphouët Boigny University, Abidjan, Côte d’Ivoire; 4 Heart Institute of Abidjan, Abidjan, Côte d’Ivoire; 5 Department of Dermatology and Infectiology, Training and Research Unit of Medical Sciences, Félix Houphouët Boigny University, Abidjan, Côte d’Ivoire; 6 Department of Infectious and Tropical Diseases, University Hospital Center of Treichville, Abidjan, Côte d’Ivoire; 7 Département of Public Health, University of Lomé, Lomé, Togo; 8 Inserm Center, 1219, University of Bordeaux, Bordeaux, France; Public Library of Science, UNITED KINGDOM

## Abstract

**Background:**

Cardiovascular diseases (CVD) are the leading causes of death in the world, mainly occurring in low-and-middle income countries. The aim of this study was to estimate the prevalence of hypertension and other cardiovascular risk factors among university students at a National Polytechnic Institute in Côte d’Ivoire.

**Methods:**

A cross-sectional study was conducted among students of the National Polytechnic Institute of Côte d’Ivoire. Sample was selected using a non-probabilistic convenient sampling method. Anthropometric measurements, blood pressure and capillary blood glucose were measured. A logistic regression model allowed to determine factors associated with hypertension.

**Results:**

A total of 2,030 students, 79.7% males and 20.3% females, with a median age of 20 years (IQR = [19–22]) participated in the study. On hypertension knowledge, 96.9% (n = 1,968) of students reported having heard of hypertension; salty foods were reported by more than a third as a cause of hypertension (n = 734; 37.3%), while 114 (5.8%) and 157 (8.0%) selected tobacco and alcohol as causes of hypertension, respectively. The overall prevalence of hypertension was 6.0%, higher in males (6.8%) compared to females (2.7%) (p < 0.001). As for CVD risk factors, 148 (7.3%) were overweight or obese; 44.0% of males and 36.6% of females reported alcohol consumption. In multivariate analysis, being a female (OR = 4.16; CI 95% = [1.96–9.09]; p<0.001), being 25 years old and older (OR = 3.34; CI 95% = [2.01–5.55]; p = 0.001), tobacco use (OR = 2.65; CI 95% = [1.41–4.96]; p = 0.002), being overweight or obese (OR = 3.75; CI 95% = [2.13–6.59]; p<0,001) and having abnormal waist circumference (OR = 6.24; CI 95% = [1.99–19.51]; p = 0.002) were significantly associated with high blood pressure.

**Conclusion:**

CVD risk factors are prominent among young adults in Côte d’Ivoire. Appropriate behavioural health interventions promoting a healthy lifestyle for young adults should be urgently implemented for CVD burden reduction.

## Introduction

Cardiovascular diseases (CVDs), a group of disorders of the heart and blood vessels, are the leading cause of death in the world. Each year, 17.9 millions of people die from CVD, accounting for about 31% of all deaths worldwide. Of these deaths, 85% are due to heart attack and stroke [1]. Currently, trends indicate a decrease in the burden of CVDs in developed countries [2] while more than 75% of CVD-related deaths occur in low and middle income countries [1]. Low awareness, treatment and control rates of the main risk factors for CVD explain this high death rate [3]. The growing evidence among young adults shows that trends in incident CVDs have increased or stagnated in recent decades [4].

An increase in cardiovascular risk factors has been recently reported in the young population [5]. In the past two decades, a high prevalence of risk factors for CVD, such as obesity, physical inactivity, and poor diet, has been observed among young individuals living in developed countries [4]. This is also the case in sub-Saharan Africa, particularly in Cameroon, where the prevalence of some major cardiovascular risk factors including physical inactivity, smoking, hazardous alcohol consumption, unhealthy diet is high among young adults [2]. Studies on cardiovascular risk factors in this population have shown that its profile could be different from that reported in older populations [5]. Current observations predict a new epidemic of CVD in this young segment of the population as it ages [4]. Therefore, specific actions should be undertaken in this young population to mitigate the upcoming burden of CVD which can be prevented by addressing the four behavioral risk factors that drive the global CVD epidemic: tobacco use, harmful use of alcohol, unhealthy diet and physical inactivity [6]. To counter the global threat of CVD, effective and evidence-based interventions must be undertaken with younger populations to delay or prevent the onset of CVD in adulthood.

However, the successful implementation of these interventions requires a more detailed knowledge of the CVD risk factors distribution in the young population. This study aimed at estimating the prevalence of the main risk factors for CVD in young students of a national university in Côte d’Ivoire, West Africa (National Polytechnic Institute of Yamoussoukro).

## Method

### Study design and setting

A cross-sectional study was conducted in a national university in Yamoussoukro, the political capital of Côte d’Ivoire, from November to December 2017, among the nine major schools of the university, with students coming from all over the country.

### Participants

Students aged 18 years and above, registered at one of the nine major schools of the institute during the 2017–2018 academic year, and who gave their consent to participate in the study were included. Participants were consecutively recruited during the study period, using a non-probabilistic convenient sampling method.

### Sample size

For calculation of the sample size, according to the results from previous studies conducted in Ethiopia, the prevalence of hypertension would be around 8% [7]. Considering a confidence interval (CI) of 95%, an estimated maximum sampling error of 1% and a 2% precision, based on the formula n = z^2^ p(1-p)/α2 (with z = confidence level; p = estimated proportion of hypertension in Ethiopia and Togo; α = precision level) and taking into account an assumption of 10% of possible missing data and refusals, the minimum sample size was estimated at 786 university students.

### Procedure for collection of data and data collection tool

A standardized, anonymous and previously tested questionnaire served for data collection. This questionnaire was elaborated by a multidisciplinary team including epidemiologists, cardiologists and members of NGOs involved in hypertension and non-communicable diseases control in Côte d’Ivoire. It comprised three sections: sociodemographic characteristics, medical history (personal and family medical history, lifestyle habits), and anthropometric and blood pressure (BP) measurements.

After obtaining informed consent, this standardized questionnaire was administered face-to-face to each university student by trained research staff.

BP measurements were taken in accordance with the “Pan-African Society of Cardiology” (PASCAR) guidelines in 2014 [8], by using an electronic sphygmomanometer (Omron M5-1, Omron Healthcare, Kyoto, Japan) on both arms. Before taking BP, each participant rested for at least 10 minutes, sitting in a chair with arms resting on an armrest, feet on the floor and back resting in the chair. BP was considered high when it was greater than 140 mmHg for systolic and / or 90 mmHg for diastolic. The measured BP were classified by grade. Thus, Systolic blood pressure (SBP)<140 mmHg and/or Diastolic blood pressure (DBP)< 90mmHg was normal, SBP = 140–159 and/or DBP = 90–99 mmHg was grade 1, SBP = 160–179 and/or DBP = 100–109 mmHg was grade 2 and SBP ≥ 180 and/or DBP ≥110 mmHg was grade 3 [9]. When BP was high on at least one of the two arms, a second measurement was done on both arms after 15 min interval. When this was too high, a third measurement was made one week later [10]. Those who had persistent high BP after all three measurements were referred to the medical service of the university for follow-up.

Bathroom scale (Numérique Verre Électronique LCD Pesant Balance De Corps 150kg New FR) was used to measure weight while height was measured using a standard rigid stadiometer. Body mass index (BMI) was calculated by dividing the weight (kg) with the height (m^2^) and grouped into the four standard categories approved by the World Health Organization (WHO): underweight (<18.5), normal (18.5–24.9), overweight (25.0–29.9) or obese (≥30.0) [11].

Mid-upper waist and hip circumferences were measured using a non-stretchable measuring tape. The waist circumference was measured at the midpoint between the top of the iliac crest and the lower margin of the last palpable rib in mid-axillary line. The hip circumference was measured at the largest circumference of the buttocks [12]. Then, the waist-to-hip ratio was calculated by dividing the waist circumference (cm) by the hip circumference (cm). Abdominal obesity was defined by a waist circumference upper than 80 centimeters for females and 94 centimeters for males [12].

### Statistical analysis

Data were coded, entered, and cleaned using Microsoft Access 2013 database (Microsoft Corporation, Redmond, WA) developed for this purpose. Data analysis was performed using SAS V.9.4 (SAS Institute, Cary, NC, USA). Results were mainly presented as median (IQR) for quantitative variables and frequency (percentage) for categorical variables. Kruskall-Wallis and Wilcoxon nonparametric tests were used to compare medians for quantitative variables. Chi-square or Fisher exact tests were used to compare categorical variables.

### Ethical consideration

This study was approved by the National Ethics Committee of Health and Life Sciences of Côte d’Ivoire and the permission of the university Directorate and of all the managers of the major schools of the Institute was granted. For each participant, written informed consent was obtained before participation in the study.

## Results

### Sociodemographic characteristics

The final sample consisted of 2,030 students (79.7% of the students were men and 20.3% were women). The median age was 20 years (interquartile range [IQR] = 19–22). Half of the students (50.3%) were in the age group of 18 to 20 years, 39.1% between 21 and 24 years old and 10.6% were 25 years old and over. The majority of participants lived at the University campus (n = 1975; 97.3%). Participants were distributed into nine major schools: Graduate School of Mines and Geology (n = 612; 30.2%), Higher School of Industry (n = 572; 28.2%), Graduate School of Business Administration (n = 324; 16.0), Higher School of Public Works (n = 201; 9.9), School of Agronomy (n = 191; 9.4), Polytechnic Doctoral School (n = 47; 2.3), Preparatory Schools (n = 43; 2.1), Regional Center for Higher Education in Metrology (n = 26; 1.2%) and international data science (n = 14; 0.7) (Table 1).

**Table 1:** Sociodemographic characteristics and lifestyle habits among university students of the National Polytechnic Institute of Côte d’Ivoire, 2017.

* *	All population		Male		Female		p-value
	*N*	*%*	*n*	*%*	*N*	*%*	
**Age (years)**							<0.001
Médian [IQR]	20 [19–22]		21 [19–22]		20 [19–21]		
**Age group (year)**							<0.001
18–20	1022	50.3	764	47.3	258	62.6	
21–24	793	39.1	664	41.0	129	31.3	
> = 25	215	10.6	190	11.7	25	6.1	
**Living with**							0.317
University campus	1975	97.3	1570	97.1	405	98.3	
with familly	31	1.5	26	1.6	5	1.2	
Alone/others	23	1.2	21	1.3	2	0.5	
**Level of study**							0.224
1st year	489	24.1	398	24.6	91	22.0	
2^nd^ year	458	22.6	361	22.3	97	23.5	
3^rd^ year	670	33.0	517	32.0	153	37.0	
4^th^ year	244	12.0	200	12.4	44	10.7	
5^th^ year	158	7.8	133	8.2	25	6.1	
other	11	0.5	8	0.5	1	0.7	
**Addition of salt to meals**							0.002
No	1,194	58.8	979	60.5	215	52.2	
Yes, often/sometimes	835	41.2	638	39.5	197	47.8	
**Alcohol consumption**							0.003
Never	1085	53.5	835	51.6	250	60.7	
Yes, but i stopped	82	4.0	71	4.4	11	2.7	
Yes, Active	863	42.5	712	44.0	151	36.6	

### Knowledge of hypertension and cardiovascular risk factors

In terms of knowledge, 96.9% (n = 1,968) of students reported having ever heard about hypertension, the majority through TV and/or radio (n = 919; 46.7%) and through friends and family (n = 795; 40.4%). Almost all participants knew that hypertension was a disease involving high BP (n = 1,613; 82%); however more than half were unable to specify the manifestations of hypertension (n = 1,086; 55.2%). Salty foods were reported by more than a third as a cause of hypertension (n = 734. 37.3%), while 114 (5.8%) and 157 (8.0%), respectively, selected tobacco and alcohol as causes of hypertension. A total of 86% of participants (n = 1,690) indicated that hypertension could be treated: 90.0% believed it could be treated by modern medicine, 53.3% by traditional medicine, 44.2% by “Chinese medicine”, and 59.2% by prayer. A total of 1,268 students (64.7%) believed hypertension could be cured, males (37.1%) more so than females (28.5%) and this difference was significant (p = .001).

### Prevalence of hypertension, anthropometric measurements and lifestyle habits

The overall prevalence of hypertension was 6.0% (n = 121), 95% CI [4.93–6.99] with a significant difference between males (6.8%) and females (2.7%) students (p < 0.001). Among them, only 64 (52.9%) returned for a third measurement one week later, and high BP was persistent for 10 (15.6%) students.

Almost all participant (98.8%) were within the normal waist circumference with a significant difference between male and female students (p < 0.001): 0.3% of males and 4.9% of females were within abnormal waist circumference (Table 2). The waist-to-hip ratio was within high values for 1.9% of males and 3.2% of females, with a significant difference (p < 0.001). On BMI classification, 127 (6.3%) were within overweight range, while 21 (1.0%) were classified as obese.

**Table 2:** Cardiovascular risk factors according to the gender among university students of the National Polytechnic Institute of Côte d’Ivoire, 2017.

* *	All population		Male		Female		p-value
	*N*	*%*	*n*	*%*	*n*	*%*	
**High BP** ^**a**^							<0.001
Normal	1,909	944.0	1,508	93.2	401	97.3	
Grade 1	104	5.1	97	6.0	7	1.7	
Grade 2	10	0.5	8	0.5	2	0.5	
Grade 3	7	0.3	5	0.3	2	0.5	
**Tobacco Use**							<0.001
Yes	51	2.5	49	3.0	2	0.5	
No	1,979	97.5	1569	97.0	410	95.5	
**BMI** ^**b**^ **(kg/m** ^**2)**^							<0.001
Underweight (<18.5)	327	16.1	246	15.2	81	19.7	
Normal (18.5–24.9)	1,555	76.6	1,293	79.9	262	63.6	
Overweight (25–29.9)	127	6.3	73	4.5	54	13.1	
Obesity (> = 30)	21	1.0	6	0.4	15	3.6	
**Waist circumference (cm)**							<0.001
M^d^: <102; W^e^: <88	2,005	98.8	1,613	99.7	392	95.1	
M : ≥102; W: ≥88	25	1.2	5	0.3	20	4.9	
**Physical activity**							0.012
Yes	1805	88.9	1453	89.8	352	85.4	
No	225	11.1	165	10.2	60	14.6	
**Cardiovascular risk factors**							0.071
None	1,362	67.1	1,105	68.3	257	62.4	
1	543	26.8	417	25.8	126	30.6	
2	112	5.5	88	5.4	24	5.8	
3	11	0.5	7	0.4	4	1.0	
4	2	0.1	1	0.1	1	0.2	

^**a**^ High BP **=** High blood pressure: Normal (SBP<140 mmHg and/or DBP < 90mmHg); Grade 1 (SBP = 140–159 and/or DBP = 90–99 mmHg); Grade 2 (SBP = 160–179 and/or DBP = 100–109 mmHg); Grade 3 (SBP ≥ 180 and/or DBP ≥110 mmHg); ^**b**^ BMI **=** Body Mass Index;^**c**^ Waist circumference: Normal (M :<102cm; W :<88cm); Abnormal (M :> = 102cm; W :> = 88cm); ^d^M: men; ^e^W: women_._

With regard to lifestyle habits, 51 (2.5%) were active smokers and 38 (1.9%) were previous smokers. Of those with active tobacco use, 5.8% consumed half a pack/year. The prevalence of alcohol active consumption was 44.0% among males and 36.6% among females (Table 1). Physical activity was practiced by 88.9% of the students with a median frequency of 2.0 [1.0–3.0] sessions per week for both men and women (p < 0.001) (Table 2).

### Association of cardiovascular risk factors

Cardiovascular risk factors apart from hypertension and including obesity, abdominal obesity, smoking and physical inactivity had been reported among some students in our study. Those had at least one cardiovascular risk factor were 543 (26.8%) and there was not significant difference between male (25.8.6%) and female (30.6%) students (p = 0.071). Participants who had two or more cardiovascular risk factors were 125 (6.1%) (Table 2).

### Multivariate analysis

In multivariate analysis, being a female (OR = 4.16; 95% CI = [1.96–9.09]; p<0.001), being 25 years old and older (OR = 3.34; 95% CI = [2.01–5.55]; p = 0.001), tobacco use (OR = 2.65; 95% CI = [1.41–4.96]; p = 0.002), being overweight or obese (OR = 3.75; 95% CI = [2.13–6.59], p<0,001) and having abnormal waist circumference (OR = 6.24; 95% CI = [1.99–19.51]; p = 0.002) were significantly associated with high BP (Table 3).

**Table 3:** Multivariate analysis of high BP among university students of the National Polytechnic Institute of Côte d’Ivoire, 2017.

	High BP^a^		Univariable analysis			Multivariate analysis		
	N	(%)	aOR^b^	(95% CI)^c^	p-value	aOR^b^	(95% CI)^c^	p-value
**Sex**					0.002			<**0.001**
Male	1,618	(6.8)	1					
Female	412	(2.7)	0.38	[0.20–0.71]		4.16	[1.96–9.09]	
**Age (years)**					<0.001			<**0.001**
18–20	1,022	(4.0)	1					
21–24	793	(6.1)	1.54	[1.01–2.36]		1.37	[0.88–2.13]	
25 and more	215	(14.9)	4.18	[2.57–6.82]		3.34	[2.01–5.55]	
**Tobacco Use**					<0.001			**0.002**
No	1,941	(5.5)	1					
Yes	89	(15.7)	3.20	[1.75–5.85]		2.65	[1.41–4.96]	
**Alcohol Consumption**					0.150	-	-	-
No	1,085	(5.3)	1					
Yes	945	(6.8)	1.31	[0.91–1.89]				
**BMI**^**d**^ **(kg/m**^**2**^**)**					<0.001			**<0.001**
18–25	1,555	(5.3)	1					
Underweight (<18.5)	327	(4.0)	0.73	[0.40–1.33]		0.92	[0.50–1.69]	
Obese / Overweight (>25)	148	(16.9)	3.60	[2.22–5.85]		3.75	[2.13–6.59]	
**Waist circumference**^e^ **(cm)**								**0.002**
M^f^: <102; W^g^: <88	2,005	(5.7)	1					
M : ≥102; W: ≥88	25	(28.0)	6.46	[2.64–15.77]		6.24	[1.99–19.51]	
**Physical activity**					0.097			
Yes	1805	(5.7)	1					
No	225	(8.4)	1.54	[0.92–2.57]		-	-	-

^**a**^ High BP = High blood pressure

^b^ aOR: adjusted odds ratio

^c^ 95% CI: 95% Confidence Interval

^**d d**^BMI **=** Body Mass Index

^**e**^ Waist circumference: Normal (M :<102cm; W :<88cm); Abnormal (M :> = 102cm; W:> = 88cm)

^f^ M: men

^g^ W: women.

## Discussion

In this study, prevalence of hypertension among young University students was 6.0% and more than a third of the sample were regular alcohol consumers. In addition, approximately one in four students had at least one CVD risk factors and almost all of them were aware of hypertension.

Knowledge and awareness of CVD, its risk factors and treatment are important elements of CVD prevention among young adults. The description of knowledge of hypertension in our study revealed that overall, university students for the majority are aware of hypertension, know its causes and consequences. In a study conducted in Malaysia, knowledge of the respondents regarding hypertension was good. This might be because almost all of the respondents got much information about hypertension from various sources, such as other people and media [13].

On the question of treatment of hypertension, more than half (59.2%) of the students believed that hypertension could be treated by prayers and approximately a third (35.3%) believed hypertension could be cured. Those beliefs on hypertension among students could undermine prevention efforts focusing on behavior changes and an emphasis on healthy lifestyle for the prevention of hypertension. This reveals that among youth, the perceived threat of the disease should be presented to heighten their beliefs on the seriousness of hypertension and to insist on the benefits of preventing it. Ignorance regarding this disease can lead to bad attitude that could be directly linked to the core of the rising hypertension prevalence as the effect of poor practice of healthy diet and lifestyle [14, 15].

The prevalence of hypertension among university students in our study corroborates with that of other studies in low-and-middle income countries. A time-series study conducted in Cameroon from 2009 to 2012 among 2,726 young students indicated a prevalence of hypertension of 6.3% [16]. A similar result was reported in the Democratic Republic of Congo; the prevalence of hypertension was estimated at 7.6% among 780 students of the university of Kikwit between January and March 2016 [17]. In nine other low-and-middle income countries of Asia (Indonesia, Laos, Myanmar, Philippines, Thailand and Vietnam) a study among 4,649 university students found that 6.7% of them had hypertension, and 19.0% had pre-hypertension [18]. A systematic review and meta-analysis on undiagnosed hypertension in sub-Saharan Africa indicated a predictive prevalence of hypertension of 16% among individuals with mean age of 30 years old [19]. Genetic factors could explain this elevated prevalence in this population and need to be better explored [20]. Despite the seemingly low prevalence of hypertension, more attention needs to be paid in preventive behavioral actions specifically targeted towards young adults and youth.

In this study, almost half of the sample indicated to be active alcohol users (42.5%) and only 8% identified alcohol consumption as a potential cause of CVD. This was corroborated by a study among medical students of the University of Makerere in Uganda, where cardiovascular risk factors with the highest prevalence was alcohol consumption with 31.7% of the sample indicating regular alcohol consumption [21]. Similar results were also reported at the university of Kikwit in the Democratic Republic of Congo with 53.1% of alcohol consumption among the sample [17]. This high alcohol consumption could be explained by the fact that students think that alcohol makes it easier to meet other people, relaxes their social inhibitions, and helps them have more fun. These effects of college environmental factors were partly explained by social-involvement, experiential, and normative expectations: college students drank for the positive consequences, because they over-estimate the drinking of their friends, or because of other normative expectations [22]. The fact that alcohol consumption is among the primary risk factors for CVD among youth is indicative of the necessity to focus on interventions for alcohol risk reduction among youth, especially to raise awareness on the consequences of regular and excessive alcohol consumption on CVD. This could also link into university policies for psychological support, as some studies revealed the potential association between heavy drinking and depressive symptoms, with prehypertension among university undergraduate students [18].

The lack of physical activity is often cited as a primary risk factor for CVD in the general population. In Somaliland, a study reported that 52% of male and 27% of female students, were physically inactive [23]. Another study of the prevalence of major CVD risk factors among university students in Cameroon revealed the same trend with 88.9% reporting physical inactivity [2]. In our study however, 88.9% of the sample indicated being physically active, with a frequency of twice per week. This could be potentially due to the fact that the National Polytechnic Institute is a selective University with an integrated, compulsory physical activity program for students. This is an example of intervention that could be adapted to other public Universities, with the goal of keeping the university students active and making physical activity a behavioral habit they could weave into their routine for the rest of their lives. On the other hand, this could also be an indication that other risk factors should also be prioritized in the prevention of CVD among young adults’ populations.

Excessive salt intake causes high BP and cardiovascular diseases. However, salt consumption among students is poorly explored. In this study, 41.5% of the sample revealed often adding more salt to their meals and only 37.3% indicated that eating “too salty” was a potential cause of hypertension. Another study in Uganda indicated that excessive salt intake was among the highest CVD risk factors among University students (13.5%) [21]. In another study, 96.7% of university students consumed over 5g /day of salt and only 6.5% of them were aware of their excessive salt intake [24]. Nutrition counseling during university registration and orientation could be given to university students and integrated into a package of healthy lifestyle interventions for students at the beginning of each academic year. The World Health Organization (WHO) strongly recommended to reduce dietary salt intake as one of the top priority actions to tackle the global non-communicable disease crisis and has urged member nations to take action to reduce population wide dietary salt intake to decrease the number of deaths from hypertension, CVD and stroke [25]. Nutrition, including salt intake should be insisted upon for the prevention of hypertension among university students especially in Africa.

In the world, 1 in 4 men and 1 in 5 women had hypertension in 2015 [26]. While the rates of hypertension in males seem to be higher than in females, in our study, there was a higher burden of CVD risk factors among females compared to males: in this study, 16.7% of females were found to have more than three cardiovascular risk factors compared to 6.6% of males and this difference was statistically significant. In addition, being female was significantly associated with an elevated BP, which is a major risk factor for hypertension. In Congo, this trend was also demonstrated with females carrying a heavier burden of modifiable cardiovascular risk factors compared to men [17]. This difference could be explained by the fact that there is high prevalence of overweight or obesity according to gender. It could be important to design sex-specific health promotion interventions that could address the particular needs of females. Observations indicate that awareness levels of CVD risk factors have to be improved among university students.

To our knowledge, this is among the first studies in Côte d’Ivoire exploring the prevalence of hypertension and cardiovascular risk factors among university students. Another strength of this study is the large sample size of university students that were recruited. However, there were some limitations. Apart from hypertension, we didn’t collect data on glycemia and lipidemia for logistical and budgetary reasons. This may lead to underestimation of cardiovascular risk factors in university students. This study took place in a highly selective University, with a convenient sample, which could potentially introduce some selection bias and could affect the generalizability of this study. In addition, the cross-sectional nature of this study, combined with the fact that most questions from the survey were self-reported could induce bias of classification. Nevertheless, as University students share the same advantage of education across all universities and because the National Polytechnic Institute include students from all backgrounds, this study could be used as a primary step toward understanding risk factors associated with hypertension among university students.

Further research, specifically cohort studies should be conducted in other Universities in Côte d’Ivoire and in the West African region in order to have a better assessment of the hypertension prevalence among young adults. In addition, it would be beneficial for other studies to go further into understanding health beliefs on hypertension and CVD, in order to better tailor interventions specific to this population.

## Conclusions

Hypertension and cardiovascular risk factors are prominent among university students in Côte d’Ivoire. Alcohol consumption and salt intake are important risk factors among this population. There is an urgent need for the implementation of preventive behavioral programs promoting a healthy lifestyle for students, that would be integrated to university policies. Further research is needed to assess the prevalence and incidence of cardiovascular diseases among young adults on a national level, in order to increase advocacy for the integration of healthy lifestyle programs in universities across the country.

## Supporting information

S1 Data (XLSX)
